# Crystal structure of 5-iodo-2-methyl-3-[(4-methyl­phenyl)­sulfon­yl]-1-benzo­furan

**DOI:** 10.1107/S1600536814018510

**Published:** 2014-08-20

**Authors:** Hong Dae Choi, Uk Lee

**Affiliations:** a Department of Chemistry, Dongeui University, San 24 Kaya-dong, Busanjin-gu, Busan 614-714, Republic of Korea; b Department of Chemistry, Pukyong National University, 599-1 Daeyeon 3-dong, Nam-gu, Busan 608-737, Republic of Korea

**Keywords:** crystal structure, benzo­furan, 4-methyl­phen­yl, π–π inter­actions, C—H⋯O hydrogen bonds, I⋯I inter­actions

## Abstract

In the title compound, C_16_H_13_IO_3_S, the dihedral angle between the planes of the benzo­furan ring system [r.m.s. deviation = 0.015 (2) Å] and the 4-methyl­phenyl ring is 70.35 (5)°. In the crystal, mol­ecules are linked by pairs of π–π inter­actions between the furan and benzene rings, with centroid–centroid distances of 3.667 (3) and 3.701 (3) Å. The mol­ecules stack along the *a*-axis direction. In addition, pairs of C—H⋯O hydrogen bonds between inversion-related dimers [which generate *R*_2_^2^(10) loops] and a short I⋯I [3.7534 (3) Å] contact are observed.

## Related literature   

For a related structure and background to benzo­furan derivatives, see: Choi & Lee (2014 ▶). For further synthetic details, see: Choi *et al.* (1999 ▶).
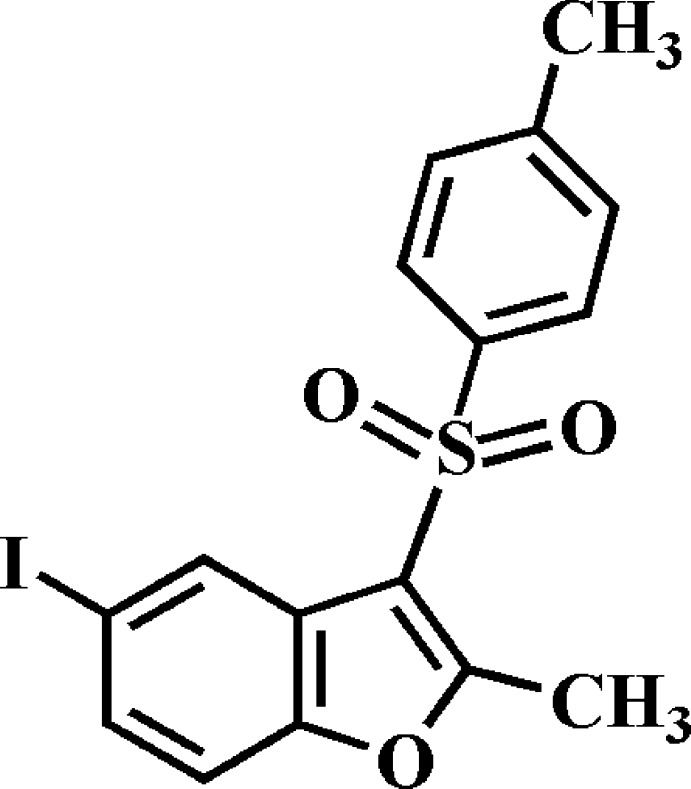


## Experimental   

### Crystal data  

C_16_H_13_IO_3_S
*M* *_r_* = 412.22
Triclinic, 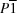

*a* = 7.2161 (1) Å
*b* = 10.5267 (2) Å
*c* = 11.3442 (2) Å
α = 111.540 (1)°
β = 90.882 (1)°
γ = 108.760 (1)°
*V* = 750.19 (2) Å^3^
*Z* = 2
Mo *K*α radiation
μ = 2.28 mm^−1^
*T* = 173 K
0.45 × 0.37 × 0.33 mm

### Data collection  

Bruker SMART APEXII CCD diffractometer
Absorption correction: multi-scan (*SADABS*; Bruker, 2009 ▶) *T*_min_ = 0.427, *T*_max_ = 0.520
13690 measured reflections
3730 independent reflections
3508 reflections with *I* > 2σ(*I*)
*R*_int_ = 0.027

### Refinement  

*R*[*F* ^2^ > 2σ(*F* ^2^)] = 0.023
*wR*(*F* ^2^) = 0.059
*S* = 1.09
3730 reflections
192 parameters
H-atom parameters constrained
Δρ_max_ = 0.53 e Å^−3^
Δρ_min_ = −0.82 e Å^−3^

### 

Data collection: *APEX2* (Bruker, 2009 ▶); cell refinement: *SAINT* (Bruker, 2009 ▶); data reduction: *SAINT*; program(s) used to solve structure: *SHELXS97* (Sheldrick, 2008 ▶); program(s) used to refine structure: *SHELXL97* (Sheldrick, 2008 ▶); molecular graphics: *ORTEP-3 for Windows* (Farrugia, 2012 ▶) and *DIAMOND* (Brandenburg, 1998 ▶); software used to prepare material for publication: *SHELXL97*.

## Supplementary Material

Crystal structure: contains datablock(s) I. DOI: 10.1107/S1600536814018510/hb7273sup1.cif

Structure factors: contains datablock(s) I. DOI: 10.1107/S1600536814018510/hb7273Isup2.hkl

Supporting information file. DOI: 10.1107/S1600536814018510/hb7273Isup3.cml

**Fig. 1.**
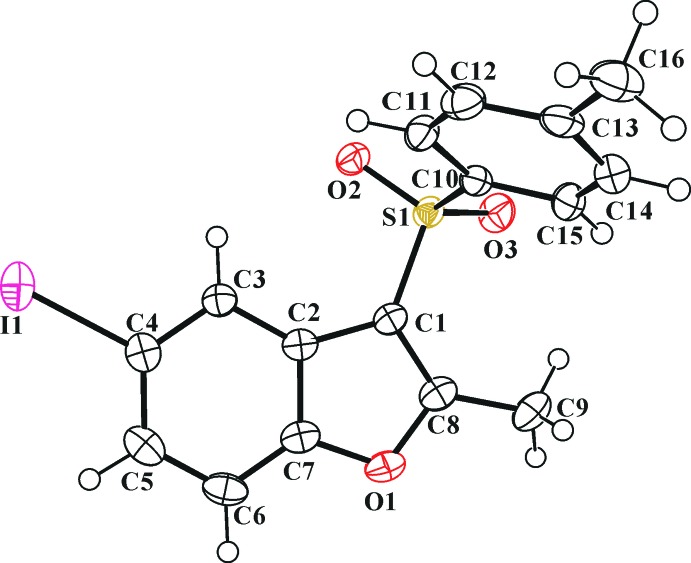
The molecular structure of the title compound with displacement ellipsoids drawn at the 50% probability level.

**Fig. 2.**
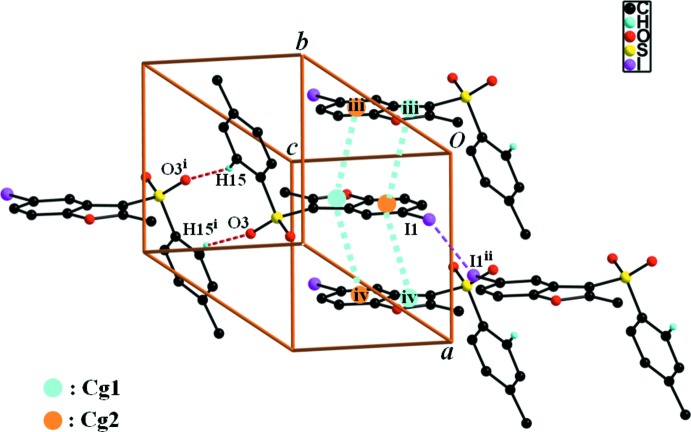
A view of the C—H···O, π–π and I···I interactions (dotted lines) in the crystal structure of the title compound. H atoms non-participating in hydrogen-bonding were omitted for clarity. [Symmetry codes: (i) - x + 1, - y + 1, - z + 2; (ii) - x + 1, - y, - z; (iii) - x, - y, - z + 1; (iv) - x + 1, - y, - z + 1.]

. DOI: 10.1107/S1600536814018510/hb7273fig1.tif
The mol­ecular structure of the title compound with displacement ellipsoids drawn at the 50% probability level.

x y z x y z x y z x y z . DOI: 10.1107/S1600536814018510/hb7273fig2.tif
A view of the C—H⋯O, π–π and I⋯I inter­actions (dotted lines) in the crystal structure of the title compound. H atoms non-participating in hydrogen-bonding were omitted for clarity. [Symmetry codes: (i) − *x* + 1, − *y* + 1, − *z* + 2; (ii) − *x* + 1, − *y*, − *z*; (iii) − *x*, − *y*, − *z* + 1; (iv) − *x* + 1, − *y*, − *z* + 1.]

CCDC reference: 1019339

Additional supporting information:  crystallographic information; 3D view; checkCIF report

## Figures and Tables

**Table 1:** Hydrogen-bond geometry (Å, °)

*D*—H⋯*A*	*D*—H	H⋯*A*	*D*⋯*A*	*D*—H⋯*A*
C15—H15⋯O3^i^	0.95	2.44	3.311 (2)	153

Symmetry code: (i) 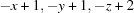.

## supplementary crystallographic information

### S1. Comment

As part of our ongoing program of benzofuran derivatives (Choi & Lee, 2014), we report herein on the crystal structure of the title compound.

In the title molecule (Fig. 1), the benzofuran unit is essentially planar, with a mean deviation of 0.015 (2) Å from the least-squares plane defined by the nine constituent atoms. The 4-methylphenyl ring is essentially planar, with a mean deviation of 0.007 (2) Å from the least-squares plane defined by the six constituent atoms. The dihedral angle formed by the benzofuran ring and the 4-methylphenyl ring is 70.35 (5)°. In the crystal structure (Fig. 2), molecules are linked by pairs of π–π interactions between the furan and benzene rings of neighbouring molecules. The molecules stack along the *a*-axis direction. The relevant centroid names for π–π stacking interactions are Cg1 for furan ring (C1/C2/C7/O1/C8) and Cg2 for the benzene ring (C2–C7). The centroid–centroid separations of Cg1···Cg2^iii^ and Cg1···Cg2^iv^ are 3.667 (3) and 3.701 (3) Å, respectively. The symmetry codes are: (iii) - *x*, - *y*, - *z* + 1; (iv) - *x* + 1, - *y*, - *z* + 1. The slippages of Cg1···Cg2^iii^ and Cg1···Cg2^iv^ are 1.379 (3) and 1.090 (3) Å, respectively. In the crystal (Fig. 2), C—H···O hydrogen bonds (Table 1) between inversion-related dimers and short I1···I1^ii^ [3.7534 (3) Å] contacts are observed.

### S2. Experimental

The starting material 5-iodo-2-methyl-3-(4-methylphenylsulfanyl)-1-benzofuran was prepared by literature method (Choi *et al.* 1999). 3-Chloroperoxybenzoic acid (77%, 448 mg, 2.0 mmol) was added in small portions to a stirred solution of 5-iodo-2-methyl-3-(4-methylphenylsulfanyl)-1-benzofuran (342 mg, 0.9 mmol) in dichloromethane (30 ml) at 273 K. After being stirred at room temperature for 10h, the mixture was washed with saturated sodium bicarbonate solution (2 × 20 ml) and the organic layer was separated, dried over magnesium sulfate, filtered and concentrated at reduced pressure. The residue was purified by column chromatography (hexane–ethyl acetate, 4:1 *v*/*v*) to afford the title compound as a colorless solid [yield 69% (284 mg); m.p. 461–462 K; *R*_f_ = 0.51 (hexane–ethyl acetate, 4:1 *v*/*v*)]. Colourless blocks were prepared by slow evaporation of a solution of the title compound (22 mg) in ethtyl acetate (10 ml) at room temperature.

### S3. Refinement

All H atoms were positioned geometrically and refined using a riding model, with C—H = 0.95 Å for aryl and 0.98 Å for methyl H atoms, *U*_iso_ (H) = 1.2*U*_eq_ (C) for aryl and 1.5*U*_eq_ (C) for methyl H atoms. The methyl groups were allowed to rotate, but not to tip, to best fit the electron density.

### Crystal data

**Table:**

C_16_H_13_IO_3_S	*Z* = 2
*M**_r_* = 412.22	*F*(000) = 404
Triclinic, *P*1	*D*_x_ = 1.825 Mg m^−^^3^
Hall symbol: -P 1	Melting point = 462–461 K
*a* = 7.2161 (1) Å	Mo *K*α radiation, λ = 0.71073 Å
*b* = 10.5267 (2) Å	Cell parameters from 9550 reflections
*c* = 11.3442 (2) Å	θ = 3.0–28.4°
α = 111.540 (1)°	µ = 2.28 mm^−^^1^
β = 90.882 (1)°	*T* = 173 K
γ = 108.760 (1)°	Block, colourless
*V* = 750.19 (2) Å^3^	0.45 × 0.37 × 0.33 mm

### Data collection

**Table:**

Bruker SMART APEXII CCD diffractometer	3730 independent reflections
Radiation source: rotating anode	3508 reflections with *I* > 2σ(*I*)
Graphite multilayer monochromator	*R*_int_ = 0.027
Detector resolution: 10.0 pixels mm^-1^	θ_max_ = 28.4°, θ_min_ = 2.0°
φ and ω scans	*h* = −8→9
Absorption correction: multi-scan (*SADABS*; Bruker, 2009)	*k* = −14→12
*T*_min_ = 0.427, *T*_max_ = 0.520	*l* = −15→15
13690 measured reflections	

### Refinement

**Table:**

Refinement on *F*^2^	Primary atom site location: structure-invariant direct methods
Least-squares matrix: full	Secondary atom site location: difference Fourier map
*R*[*F*^2^ > 2σ(*F*^2^)] = 0.023	Hydrogen site location: difference Fourier map
*wR*(*F*^2^) = 0.059	H-atom parameters constrained
*S* = 1.09	*w* = 1/[σ^2^(*F*_o_^2^) + (0.0251*P*)^2^ + 0.4508*P*] where *P* = (*F*_o_^2^ + 2*F*_c_^2^)/3
3730 reflections	(Δ/σ)_max_ < 0.001
192 parameters	Δρ_max_ = 0.53 e Å^−^^3^
0 restraints	Δρ_min_ = −0.82 e Å^−^^3^

### Special details

**Table:**

Experimental. ^1^H NMR (δ p.p.m., CDCl3, 400 Hz): 8.23 (s, 1H), 7.87 (d, J = 8.56 Hz, 2H), 7.58 (dd, J = 8.56 and 1.72 Hz, 1H), 7.32 (d, J = 8.20 Hz, 2H), 7.17 (d, J = 8.56 Hz, 1H), 2.78 (s, 3H), 2.40 (s, 3H).
Geometry. All esds (except the esd in the dihedral angle between two l.s. planes) are estimated using the full covariance matrix. The cell esds are taken into account individually in the estimation of esds in distances, angles and torsion angles; correlations between esds in cell parameters are only used when they are defined by crystal symmetry. An approximate (isotropic) treatment of cell esds is used for estimating esds involving l.s. planes.
Refinement. Refinement of F^2^ against ALL reflections. The weighted R-factor wR and goodness of fit S are based on F^2^, conventional R-factors R are based on F, with F set to zero for negative F^2^. The threshold expression of F^2^ > 2sigma(F^2^) is used only for calculating R-factors(gt) etc. and is not relevant to the choice of reflections for refinement. R-factors based on F^2^ are statistically about twice as large as those based on F, and R- factors based on ALL data will be even larger.

### Fractional atomic coordinates and isotropic or equivalent isotropic displacement parameters (Å^2^)

**Table:**

	*x*	*y*	*z*	*U*_iso_*/*U*_eq_	
I1	0.34881 (2)	0.005191 (17)	0.133697 (13)	0.03924 (6)	
S1	0.51630 (7)	0.39711 (5)	0.72563 (4)	0.02485 (10)	
O1	0.1860 (2)	−0.00618 (16)	0.66250 (15)	0.0303 (3)	
O2	0.6298 (2)	0.41551 (16)	0.62596 (13)	0.0302 (3)	
O3	0.6169 (2)	0.43950 (18)	0.85197 (14)	0.0346 (3)	
C1	0.3716 (3)	0.2132 (2)	0.66867 (18)	0.0245 (4)	
C2	0.3274 (3)	0.1121 (2)	0.53672 (18)	0.0236 (4)	
C3	0.3694 (3)	0.1210 (2)	0.41971 (19)	0.0257 (4)	
H3	0.4451	0.2108	0.4148	0.031*	
C4	0.2959 (3)	−0.0065 (2)	0.31154 (19)	0.0283 (4)	
C5	0.1880 (3)	−0.1412 (2)	0.3154 (2)	0.0329 (4)	
H5	0.1444	−0.2267	0.2386	0.040*	
C6	0.1449 (3)	−0.1498 (2)	0.4313 (2)	0.0326 (4)	
H6	0.0707	−0.2398	0.4364	0.039*	
C7	0.2144 (3)	−0.0220 (2)	0.5385 (2)	0.0268 (4)	
C8	0.2817 (3)	0.1378 (2)	0.7397 (2)	0.0281 (4)	
C9	0.2619 (3)	0.1795 (3)	0.8770 (2)	0.0365 (5)	
H9A	0.3626	0.2751	0.9271	0.055*	
H9B	0.2802	0.1066	0.9058	0.055*	
H9C	0.1299	0.1844	0.8893	0.055*	
C10	0.3497 (3)	0.4906 (2)	0.73988 (18)	0.0248 (4)	
C11	0.3006 (3)	0.5246 (2)	0.63922 (19)	0.0288 (4)	
H11	0.3530	0.4950	0.5615	0.035*	
C12	0.1741 (3)	0.6022 (2)	0.6538 (2)	0.0328 (4)	
H12	0.1422	0.6274	0.5858	0.039*	
C13	0.0926 (3)	0.6441 (2)	0.7656 (2)	0.0309 (4)	
C14	0.1398 (3)	0.6056 (2)	0.8638 (2)	0.0345 (5)	
H14	0.0825	0.6315	0.9399	0.041*	
C15	0.2688 (3)	0.5302 (2)	0.85277 (19)	0.0317 (4)	
H15	0.3016	0.5059	0.9211	0.038*	
C16	−0.0386 (4)	0.7339 (2)	0.7832 (3)	0.0412 (5)	
H16A	0.0429	0.8378	0.8215	0.062*	
H16B	−0.1335	0.7112	0.8399	0.062*	
H16C	−0.1104	0.7110	0.6998	0.062*	

### Atomic displacement parameters (Å^2^)

**Table:**

	*U*^11^	*U*^22^	*U*^33^	*U*^12^	*U*^13^	*U*^23^
I1	0.04516 (10)	0.04799 (10)	0.02485 (8)	0.02137 (7)	0.00481 (6)	0.01037 (6)
S1	0.0240 (2)	0.0273 (2)	0.0206 (2)	0.00442 (17)	0.00161 (17)	0.01065 (18)
O1	0.0292 (7)	0.0299 (7)	0.0393 (8)	0.0103 (6)	0.0085 (6)	0.0220 (6)
O2	0.0286 (7)	0.0306 (7)	0.0267 (7)	0.0034 (6)	0.0067 (6)	0.0122 (6)
O3	0.0315 (7)	0.0426 (8)	0.0248 (7)	0.0068 (6)	−0.0035 (6)	0.0135 (6)
C1	0.0223 (8)	0.0273 (9)	0.0269 (9)	0.0090 (7)	0.0047 (7)	0.0137 (8)
C2	0.0197 (8)	0.0248 (9)	0.0292 (9)	0.0091 (7)	0.0029 (7)	0.0128 (7)
C3	0.0245 (9)	0.0259 (9)	0.0277 (9)	0.0085 (7)	0.0032 (7)	0.0119 (8)
C4	0.0269 (9)	0.0314 (10)	0.0281 (10)	0.0144 (8)	0.0017 (7)	0.0100 (8)
C5	0.0312 (10)	0.0265 (10)	0.0369 (11)	0.0121 (8)	−0.0046 (8)	0.0065 (8)
C6	0.0278 (10)	0.0237 (9)	0.0472 (12)	0.0083 (8)	0.0008 (9)	0.0158 (9)
C7	0.0224 (8)	0.0287 (9)	0.0355 (10)	0.0106 (7)	0.0043 (7)	0.0182 (8)
C8	0.0256 (9)	0.0328 (10)	0.0335 (10)	0.0126 (8)	0.0057 (8)	0.0192 (8)
C9	0.0394 (12)	0.0470 (13)	0.0352 (11)	0.0175 (10)	0.0129 (9)	0.0269 (10)
C10	0.0285 (9)	0.0209 (8)	0.0216 (8)	0.0046 (7)	0.0016 (7)	0.0084 (7)
C11	0.0320 (10)	0.0302 (10)	0.0222 (9)	0.0064 (8)	0.0042 (7)	0.0120 (8)
C12	0.0351 (11)	0.0312 (10)	0.0325 (11)	0.0065 (8)	0.0004 (8)	0.0176 (9)
C13	0.0280 (9)	0.0198 (9)	0.0388 (11)	0.0024 (7)	0.0036 (8)	0.0103 (8)
C14	0.0426 (12)	0.0287 (10)	0.0300 (10)	0.0121 (9)	0.0111 (9)	0.0094 (8)
C15	0.0427 (11)	0.0307 (10)	0.0212 (9)	0.0113 (9)	0.0047 (8)	0.0111 (8)
C16	0.0402 (12)	0.0277 (11)	0.0558 (15)	0.0123 (9)	0.0077 (11)	0.0161 (10)

### Geometric parameters (Å, º)

**Table:**

I1—C4	2.099 (2)	C8—C9	1.478 (3)
I1—I1^i^	3.7534 (3)	C9—H9A	0.9800
S1—O3	1.4389 (14)	C9—H9B	0.9800
S1—O2	1.4400 (14)	C9—H9C	0.9800
S1—C1	1.737 (2)	C10—C11	1.388 (3)
S1—C10	1.758 (2)	C10—C15	1.394 (3)
O1—C8	1.369 (3)	C11—C12	1.383 (3)
O1—C7	1.381 (3)	C11—H11	0.9500
C1—C8	1.362 (3)	C12—C13	1.388 (3)
C1—C2	1.439 (3)	C12—H12	0.9500
C2—C7	1.393 (3)	C13—C14	1.389 (3)
C2—C3	1.396 (3)	C13—C16	1.507 (3)
C3—C4	1.379 (3)	C14—C15	1.384 (3)
C3—H3	0.9500	C14—H14	0.9500
C4—C5	1.398 (3)	C15—H15	0.9500
C5—C6	1.384 (3)	C16—H16A	0.9800
C5—H5	0.9500	C16—H16B	0.9800
C6—C7	1.374 (3)	C16—H16C	0.9800
C6—H6	0.9500		
			
C4—I1—I1^i^	152.81 (5)	O1—C8—C9	115.80 (17)
O3—S1—O2	119.63 (9)	C8—C9—H9A	109.5
O3—S1—C1	108.45 (10)	C8—C9—H9B	109.5
O2—S1—C1	106.07 (9)	H9A—C9—H9B	109.5
O3—S1—C10	107.93 (10)	C8—C9—H9C	109.5
O2—S1—C10	108.10 (9)	H9A—C9—H9C	109.5
C1—S1—C10	105.86 (9)	H9B—C9—H9C	109.5
C8—O1—C7	107.15 (15)	C11—C10—C15	120.7 (2)
C8—C1—C2	107.56 (17)	C11—C10—S1	119.87 (16)
C8—C1—S1	126.69 (16)	C15—C10—S1	119.40 (16)
C2—C1—S1	125.74 (14)	C12—C11—C10	118.98 (19)
C7—C2—C3	119.25 (18)	C12—C11—H11	120.5
C7—C2—C1	105.06 (17)	C10—C11—H11	120.5
C3—C2—C1	135.69 (17)	C11—C12—C13	121.5 (2)
C4—C3—C2	116.96 (18)	C11—C12—H12	119.3
C4—C3—H3	121.5	C13—C12—H12	119.3
C2—C3—H3	121.5	C12—C13—C14	118.5 (2)
C3—C4—C5	123.1 (2)	C12—C13—C16	121.3 (2)
C3—C4—I1	117.72 (15)	C14—C13—C16	120.1 (2)
C5—C4—I1	119.19 (15)	C15—C14—C13	121.3 (2)
C6—C5—C4	119.9 (2)	C15—C14—H14	119.4
C6—C5—H5	120.0	C13—C14—H14	119.4
C4—C5—H5	120.0	C14—C15—C10	118.99 (19)
C7—C6—C5	116.79 (19)	C14—C15—H15	120.5
C7—C6—H6	121.6	C10—C15—H15	120.5
C5—C6—H6	121.6	C13—C16—H16A	109.5
C6—C7—O1	126.11 (18)	C13—C16—H16B	109.5
C6—C7—C2	123.91 (19)	H16A—C16—H16B	109.5
O1—C7—C2	109.97 (17)	C13—C16—H16C	109.5
C1—C8—O1	110.23 (18)	H16A—C16—H16C	109.5
C1—C8—C9	133.9 (2)	H16B—C16—H16C	109.5
			
O3—S1—C1—C8	31.6 (2)	C3—C2—C7—O1	−178.42 (16)
O2—S1—C1—C8	161.28 (18)	C1—C2—C7—O1	1.1 (2)
C10—S1—C1—C8	−84.0 (2)	C2—C1—C8—O1	1.4 (2)
O3—S1—C1—C2	−147.56 (17)	S1—C1—C8—O1	−177.87 (14)
O2—S1—C1—C2	−17.9 (2)	C2—C1—C8—C9	−176.3 (2)
C10—S1—C1—C2	96.83 (18)	S1—C1—C8—C9	4.4 (4)
C8—C1—C2—C7	−1.5 (2)	C7—O1—C8—C1	−0.7 (2)
S1—C1—C2—C7	177.78 (15)	C7—O1—C8—C9	177.42 (17)
C8—C1—C2—C3	177.9 (2)	O3—S1—C10—C11	151.02 (16)
S1—C1—C2—C3	−2.8 (3)	O2—S1—C10—C11	20.28 (18)
C7—C2—C3—C4	−0.5 (3)	C1—S1—C10—C11	−93.02 (17)
C1—C2—C3—C4	−179.8 (2)	O3—S1—C10—C15	−28.79 (18)
C2—C3—C4—C5	−1.6 (3)	O2—S1—C10—C15	−159.52 (15)
C2—C3—C4—I1	178.02 (14)	C1—S1—C10—C15	87.17 (17)
I1^i^—I1—C4—C3	89.06 (19)	C15—C10—C11—C12	1.8 (3)
I1^i^—I1—C4—C5	−91.27 (19)	S1—C10—C11—C12	−178.00 (15)
C3—C4—C5—C6	2.3 (3)	C10—C11—C12—C13	−1.3 (3)
I1—C4—C5—C6	−177.38 (15)	C11—C12—C13—C14	−0.4 (3)
C4—C5—C6—C7	−0.6 (3)	C11—C12—C13—C16	177.49 (19)
C5—C6—C7—O1	179.13 (19)	C12—C13—C14—C15	1.5 (3)
C5—C6—C7—C2	−1.5 (3)	C16—C13—C14—C15	−176.3 (2)
C8—O1—C7—C6	179.2 (2)	C13—C14—C15—C10	−1.0 (3)
C8—O1—C7—C2	−0.3 (2)	C11—C10—C15—C14	−0.7 (3)
C3—C2—C7—C6	2.1 (3)	S1—C10—C15—C14	179.13 (16)
C1—C2—C7—C6	−178.35 (19)		

Symmetry code: (i) −*x*+1, −*y*, −*z*.

### Hydrogen-bond geometry (Å, º)

**Table:**

*D*—H···*A*	*D*—H	H···*A*	*D*···*A*	*D*—H···*A*
C15—H15···O3^ii^	0.95	2.44	3.311 (2)	153

Symmetry code: (ii) −*x*+1, −*y*+1, −*z*+2.
