# Supplementary figures and images for: Crystal structure of 5-iodo-2-methyl-3-[(4-methyl­phenyl)­sulfon­yl]-1-benzo­furan
Source: Acta Crystallogr Sect E Struct Rep Online. 2014 Aug 20;70(Pt 9):o1028. doi: 10.1107/S1600536814018510 (PMC4186123; doi:10.1107/S1600536814018510)

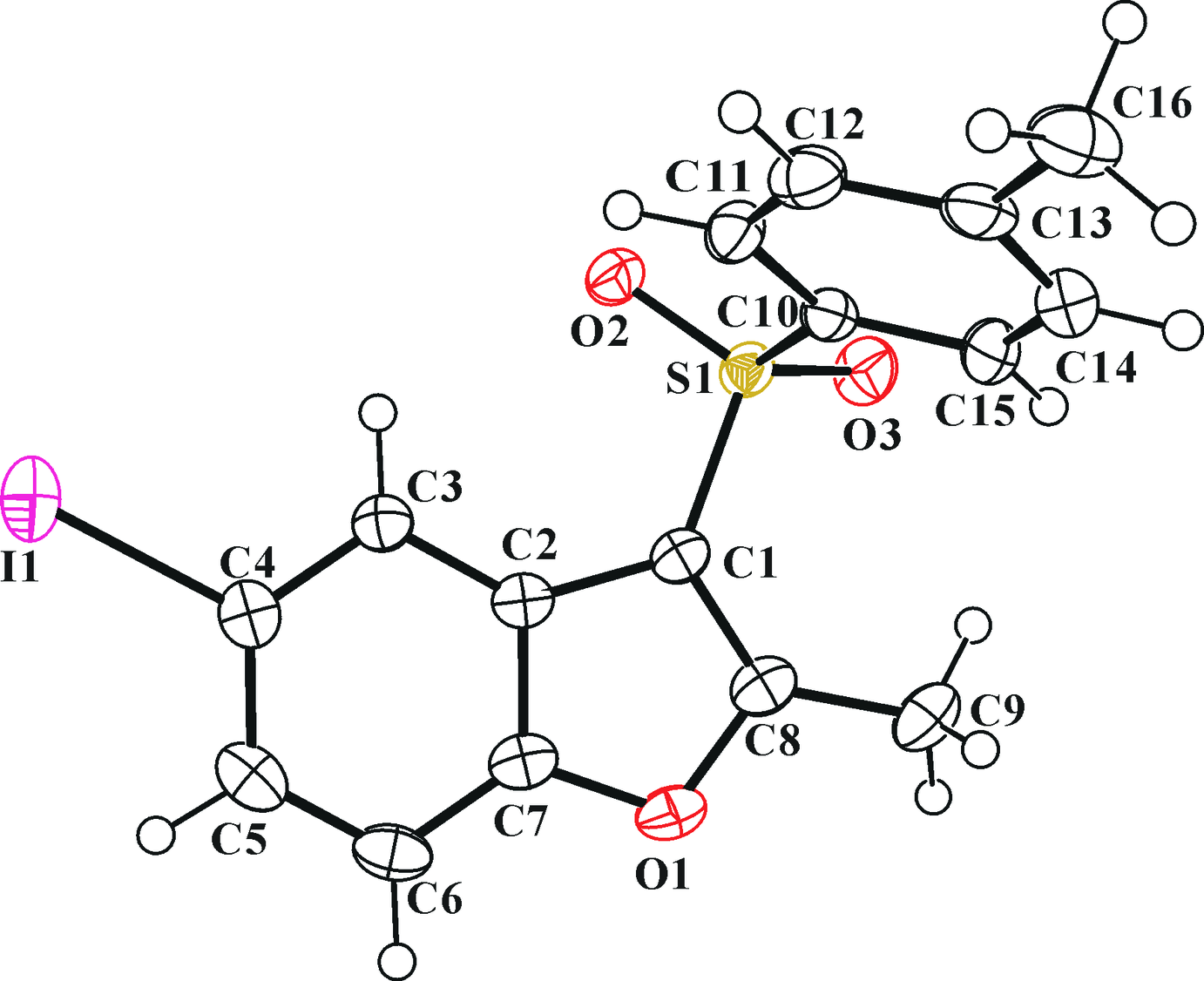

Supplement: Supplementary file 4 [file e-70-o1028-fig1.tif]

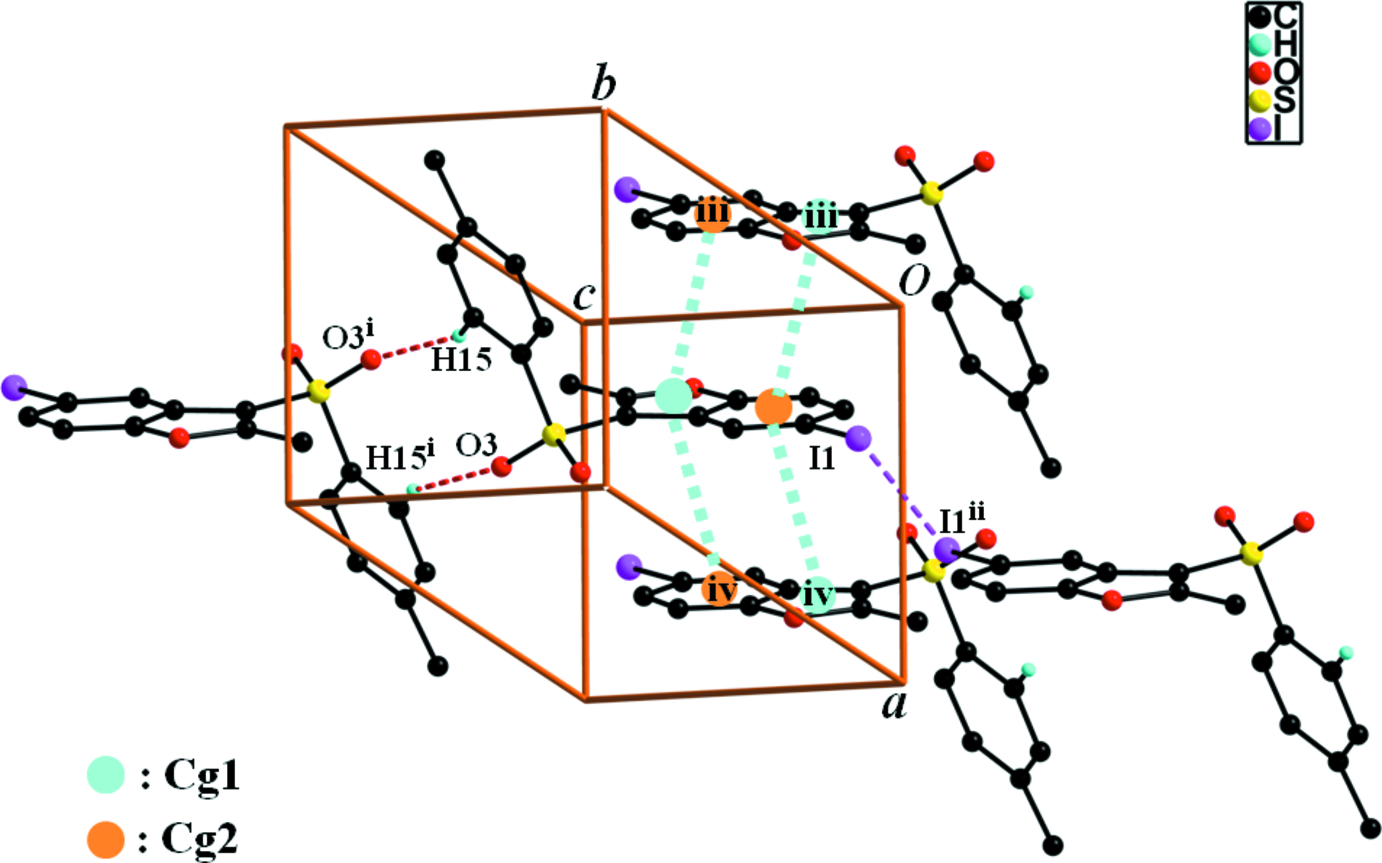

Supplement: Supplementary file 5 [file e-70-o1028-fig2.tif]
